# Xeno-Hybrid Bone Graft Releasing Biomimetic Proteins Promotes Osteogenic Differentiation of hMSCs

**DOI:** 10.3389/fcell.2020.619111

**Published:** 2020-12-22

**Authors:** Hao Zhu, Veronika Hefka Blahnová, Giuseppe Perale, Jun Xiao, Felice Betge, Fabio Boniolo, Eva Filová, Ståle Petter Lyngstadaas, Håvard Jostein Haugen

**Affiliations:** ^1^Department of Orthopedic Surgery, Tongji Hospital, Tongji Medical College, Huazhong University of Science and Technology, Wuhan, China; ^2^Department of Tissue Engineering, Institute of Experimental Medicine of the Czech Academy of Sciences, Prague, Czechia; ^3^Department of Biophysics, Second Faculty of Medicine, Charles University, Prague, Czechia; ^4^Industrie Biomediche Insubri S.A., Mezzovico-Vira, Switzerland; ^5^Ludwig Boltzmann Institute for Experimental and Clinical Traumatology, Vienna, Austria; ^6^Faculty of Biomedical Sciences, University of Southern Switzerland, Lugano, Switzerland; ^7^Helmholtz Zentrum München Deutsches Forschungszentrum für Gesundheit und Umwelt (GmbH), Neuherberg, Germany; ^8^Corticalis AS, Oslo, Norway; ^9^Department of Biomaterials, Faculty of Dentistry, University of Oslo, Oslo, Norway

**Keywords:** bone scaffold, bone graft, bone regeneration biomimetic, bioactive proteins, intrinsically disordered, mesenchymal stem cells, xenograft

## Abstract

Bone defect is a noteworthy health problem and is the second most transplanted tissue after blood. Numerous bone grafts are designed and applied in clinics. Limitations, however, from different aspects still exist, including limited supply, mechanical strength, and bioactivity. In this study, two biomimetic peptides (P2 and P6) are incorporated into a composite bioactive xeno hybrid bone graft named SmartBonePep^®^, with the aim to increase the bioactivity of the bone graft. The results, which include cytotoxicity, proliferation rate, confocal microscopy, gene expression, and protein qualification, successfully prove that the SmartBonePep^®^ has multi-modal biological effects on human mesenchymal stem cells from bone marrow. The effective physical entrapment of P6 into a composite xeno-hybrid bone graft, withstanding manufacturing processes including exposure to strong organic solvents and ethylene oxide sterilization, increases the osteogenic potential of the stem cells as well as cell attachment and proliferation. P2 and P6 both show a strong biological potential and may be future candidates for enhancing the clinical performance of bone grafts.

## Introduction

Trauma, surgical resection, infections, degeneration, and a myriad of external and internal factors can alone or in combination lead to critical-size bone defects. Such defects are severe public health issues and take impact on public healthcare and on the quality of life of the patients involved (Wang and Yeung, 2017). In fact, disorder of musculoskeletal system is a familiar reason of long-term discomfort. Although skeletal tissues have certain capacity of self-healing, it remains limited in critical-size defects where callus formation is unable to bridge and stabilize the compromised bone. This situation often results in non-union fractures, the formation of pseudarthroses or skeletal deformation (Winkler et al., 2018). In order to avoid such complications an implant and/or a graft is often used in a surgical procedure to stabilize the bone and induce new bone formation *in situ*. Bone grafts and bone graft substitutes play an important role in terms of mechanically supporting the defect, attracting cells, guiding bone ingrowth, and inducing bone regeneration (Reczyńska et al., 2015; Ionita et al., 2016; Winkler et al., 2018). Even though a wide range of synthetic biomaterials with diverse characteristics are available for clinical use today, e.g., titanium alloys or calcium phosphates (Campana et al., 2014; Cama et al., 2017; Sukul et al., 2020)[Bibr B2][Bibr B3][Bibr B49], there are still important limitations in their biological aptness (Rahmati et al., 2020). This includes poor or non-predictable biodegradation rates (Rumpel et al., 2006), or lack of osteogenic capacity (Esposito et al., 1999; Sakka and Coulthard, 2011). Therefore, natural bone grafts are still of great interest. Autologous bone grafts were the first grafts used to repair bone defects and have shown great success (Giannoudis et al., 2005). This type of technique does, however, requires additional surgery, e.g., osteotomy on the iliac, causes extra pain and is linked to donor site morbidity, which represents an additional risk especially for the older population. More importantly, autologous bone sources are limited, and the quality of the autograft is in some cases not high enough (Haugen et al., 2019). Allogeneic bone graft, bone from different and often dead human donors, is another option for bone repair. This material allows the treatment of larger defects, the only manufacturing limitations being amount, type, and qualities. Nevertheless, the number of potential donors is becoming increasingly limited and ethical concerns are rising. Moreover, allogeneic transplants and grafts also involve potential risks such as triggering immunogenic reactions or transferring contagious diseases (Sohn and Oh, 2019). An appropriate bone substitutes ought to resemble the naturally bone, xenografts, i.e., bone tissue from another species, are also considered to be a viable option, and to be on a par with allografts and synthetics (Athanasiou et al., 2010; Schmitt et al., 2015). Amongst commonly used xenografts, bovine cancellous bone grafts are regarded the most similar to human bone (Poumarat and Squire, 1993; Fletcher et al., 2018). Albeit sharing the risk profile with allografts, they are more widely used because they are cheap, easily available in large amounts, and are standardized. Their safety and efficacy in skeletal reconstructive surgery since the 1950s has also been demonstrated (Planell et al., 2009).

Most xenografts are produced by defatting, decellularizing, and deproteinizing cancellous bone, which are necessary processes for the elimination of antigenicity, the mineralized bone matrix alone being the substance in clinical use (Meloni et al., 2017). Thus, the toughness of xenograft is lowered compared to natural bone even though the strength can be mostly retained (Cornu, 2012). Moreover, due to the process of sterilization and storage allografts and xenografts have significantly decreased osteogenic and osteoinductive function compared to living autografts (Pape et al., 2010). A new concept using a xeno-hybrid graft substitute has been introduced to reduce this discrepancy between xenografts and natural bone. This medical device, named SmartBone^®^ and certified for clinical use, is manufactured by adding a biocompatible polymer coating and gelatine-derived collagen fragments to the deproteinized bovine bone matrix, in order to create a biomaterial that has similar characteristics to natural bone (Pertici et al., 2014; D’Alessandro et al., 2017; Cingolani et al., 2018; Ferracini et al., 2019). The polymer used poly(L-lactide-co-ε-caprolactone) (PCL-LA) increases the toughness of the graft, while the collagen fragments present the integrin binding motif, RGD, on the polymeric coating with the specific role to further improve the biological performance of the biomaterial (Rossi et al., 2015; Roato et al., 2018).

Bioactive macromolecules such as growth factors or cell attachment motifs, have been suggested to be favorable for improved bone regeneration in critical-size bone defects. The RGD motif is widely used to promote initial mesenchymal cell attachment onto scaffolding materials via integrin receptors (Kapp et al., 2017; Chahal et al., 2020). However, cell attachment in itself is not enough for optimized bone repair, while the following proliferation and differentiation process of the attached cells needs further stimulation from the surrounding microenvironment to enable proper bone repair. Growth factors and other biomolecules can be utilized to accelerate bone healing when incorporated into a bone graft substitute (Lv et al., 2015; Lin et al., 2019). Several biomolecules have obtained FDA approval for such use including BMPs (bone morphogenetic proteins), bFGF (basic fibroblast growth factor), and VEGF (vascular endothelial growth factor) (Janicki and Schmidmaier, 2011; Wang and Yeung, 2017). The regulation of bone healing by a growth factor is, however, a very complex and highly orchestrated process that need to be closely controlled at several stages to avoid deleterious treatment outcomes. Thus, there is intense controversy regarding the clinical use of growth factors such as BMPs, and hence a pressing need to find safer and more effective bone stimulating strategies for bone tissue engineering that better biomimics the natural healing process (Epstein, 2013; Oryan et al., 2014).

This study aims to investigate the potential of biomimetic peptides, belonging to a group of proteins called “intrinsically disordered proteins” (IDPs), which are highly diverse in their effects and yet adaptable and specific, in their action. These molecules have no strict internal structure and can therefore adapt to several binding configurations due to their one-to-many and many-to-one signaling capacities (Wright and Dyson, 2015). These two biomimetic peptides P2 and P6 were fused into the polymeric coating of the SmartBone^®^ matrix, simultaneously with the RGD motif during the graft production process, in an effort to further increase the bio-efficacy of this promising xeno-hybrid bone graft substitute. The peptide sequence P6 is identical to part of the amelogenin (AMEL) sequence (Zhang et al., 2010; Wald et al., 2011). Amelogenin naturally occurs in tooth enamel and plays a major role in matrix biomineralization (Fang et al., 2011; Ruan and Moradian-Oldak, 2015). P2 biomimetic nature is based on the common characteristics of proline-rich regions in hard tissue extracellular matrix proteins (Rubert et al., 2011; Villa et al., 2016). Another main advantageous of these biomimetic peptides is that they can withstand organic solvents and thus is more versatile when incorporation into new hybrid biomaterials than growth factors (e.g., BMPs).

Since bone marrow derived human mesenchymal stem cells (hMSCs) are the most abundant progenitor cell-type present at the site of bone injury and are the major facilitators of bone regeneration, we tested the biocompatibility and bio-efficacy of these novel composite bone grafts in hMSCs cultures, both growth culture medium and differentiation medium with osteogenic supplements being used. The effects of bone graft with added biomimetic proteins P2 and P6 on hMSCs were assessed by cytotoxicity assays, proliferation assays, real-time PCR, multiplex proteomics, immunofluorescence staining, and confocal microscopy.

## Materials and Methods

### Bone Graft Preparations

**FIGURE 1:**
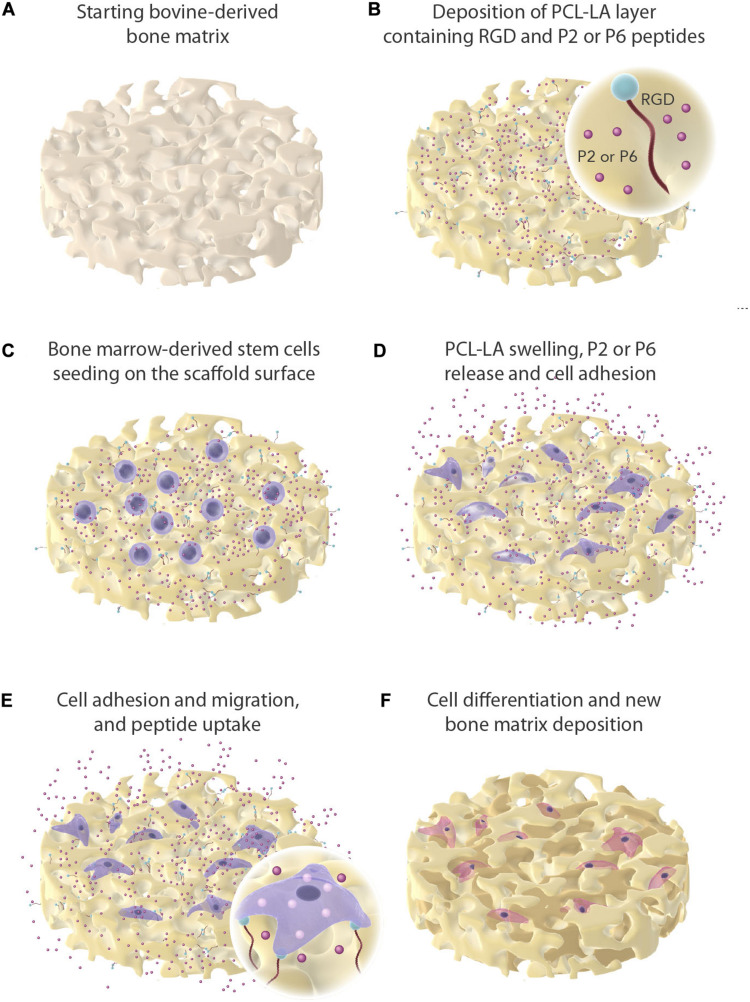
Graphical overview of experimental work. Dissolved and physical entrapment of P2 and P6 in a polymer matrix (PCL-LA) and mesenchymal stem cells seeded onto the bone graft. The peptides are presented as red sphere and are being released off the bone graft as the PCL-LA swells.

The xeno-hybrid bone graft SmartBone^®^ (SBN) was manufactured by I.B.I. SA (Mezzovico-Vira, Switzerland). It consists of a bovine bone-derived mineral matrix which is improved by reinforcement with the co-polymer coating poly(L-lactide-co-ε-caprolactone) (PLCL) and the addition of RGD-exposing collagen fragments from animal-derived gelatine (D’Alessandro et al., 2017). During standard manufacturing process of SBN, the biomimetic peptides P2 and P6 (Zhu et al., 2020) were embedded into the polymer coating of SBN, producing SmartBonePep^®^ (SBP) with a nominal concentration capable to provide a release rate equivalent to 1 μg/cm^3^/d over a 2 weeks time (Figures 1A–F) (Perale et al., 2019). These two bone grafts were called SBP2 and SBP6, respectively (Zhu et al., 2020). A third group which combine both P2 and P6 added was also added (SBP2+P6). The release profiles, cytotoxicity, SEM, and mechanical strength of both SBN and SBP has been described by Perale et al. (2019) and thus not repeated here. The sequences of the biomimetic synthetic peptides P2 and P6 (designed by Corticalis AS, Oslo, Norway and supplied by Pepmic Co., Ltd., Jiangsu, China) are available are previously described (Ramis et al., 2012). Both peptides were prepared as stock solutions at 4 mM in 0.1% acetic acid and stored at −20°C.

### Cell Culturing, Expansion, and Seeding

Commercially available hMSCs (Lonza, Germany) were employed for cell experiments. Cells were cultured in mesenchymal stem cell growth medium (MSCGM, Lonza, Germany) as recommended by the cell provider, in a standard cell culturing environment of 37°C humidified atmosphere with 5% CO_2_. Two types of medium were used in the experiments: (1) growth medium and (2) differentiation medium. The osteogenic differentiation medium was made by the enrichment of basal medium with 10 mM β-glycerophosphate (50020, Sigma Aldrich) and 100 μM ascorbate-2-phosphate (A8960, Sigma Aldrich). For the cell expansion, the growth medium was used and changed every 3 days, and cells between passage 5 and 7 were used for experiments. If not specified, differentiation medium was used for experiments. A suspension of 8 × 10^4^ cells, volume of 200 μl, was microseeded on the surface of three different samples per group (circular shaped, diameter: 12 mm, height: 3 mm) in a 24-well plate and redone three times (three replicates). After 30 min incubation for cell attachment and penetration inside the graft matrix by media and cells soaking (Roato et al., 2018), full volume of the culturing medium was then gently added. The medium was changed on day 2, 5, and 7 of each week and cells were cultured until day 28 at 37°C and 5% CO_2_.

### Cytotoxicity

Following indications by current revisions of the ISO-10993 norm series, lactate dehydrogenase (LDH) activity in the culture media was used to evaluate the cytotoxicity of the samples (four samples, replicated three times, *n* = 12). All the measurements were performed according to the manufacturer’s kit instructions (Roche Diagnostics, Mannheim, Germany). After 48 h of culturing, 50 μl of the culture medium was taken out and mixed with 50 μl of the reaction mixture. The incubation was performed at room temperature for 30 min in the dark environment. LDH activity was determined in an ELISA reader by measuring the oxidation of nicotinamide adenine dinucleotide (NADH) at 490 nm in the presence of pyruvate. Results were presented relative to the control by calculating the OD value as follows. Cells cultured in the culturing plate without interference were set as the negative control, while cells in the culturing plate treated with medium containing 1% Triton X-100 were set as the positive control (100% cell death).

Cytotoxicity was calculated using the following equation (Eq. 1):

(1) $$\text{Cytotoxicity}=\left(\frac{\mathrm{Experimental}\mathrm{group}-\mathrm{Negative}\mathrm{control}}{\mathrm{Positive}\mathrm{control}-\mathrm{Negative}\mathrm{control}}\right)\times 100\%$$

### Cell Viability/Metabolic Rate Measurement

The MTS test was used for cell viability evaluation. Samples were transferred to a new 24-well plate to avoid measuring the metabolic activity of the cells that had adhered to the tissue culture plastics. The test was run on three replicates. 120 μl of MTS substrate (CellTiter 96^®^ AQueous MTS Reagent Powder G1111, Promega) with 600 μl of culture medium was added to each sample. Thereafter, the samples were incubated at 37°C and 5% CO_2_ for 2 h. During this time the yellow substrate was reduced by mitochondrial enzymes to a purple product. The product absorbance was measured using the reader InfiniteM200PRO (Tecan) at 490 nm, reference wavelength 690 nm.

### Cell Proliferation Assay

The samples (*n* = 3) after performing MTS test were washed twice with PBS and transferred to sterile tubes with 700 μl of lysis buffer in each. The lysis buffer consists of 10 mM Tris (T1503, Sigma Aldrich), 1 mM EDTA (EDS, Sigma Aldrich) with 0.0004% Triton X-100 (T8787, Sigma Aldrich). Samples were then frozen. They were vortexed after thawing. Samples were further frozen, thawed, and vortexed, three cycles in total. Finally, 10 μl of sample, respectively dsDNA standard from the assay, was transferred to a black 96-well plate with a transparent bottom. Then 200 μl of working solution Quant-iT^TM^ dsDNA Assay Kit (Q33120, Invitrogen) was added. A fluorescently labeled probe in the working solution emitted a signal after binding to dsDNA. The fluorescence was measured at excitation wavelength 485 nm and emission 528 nm.

### Confocal Microscopy

Confocal microscopy was performed on three samples for each group at each time point on day 14th and 21st to observe cell expansion. Samples were first washed three times with PBS and then fixed with 4% paraformaldehyde for 12 min. After washed with PBS for three times, samples were stained with phalloidin labeled with Alexa-488 for 1 h and DAPI for 20 min. Original images were taken using laser scanning confocal microscopy (Leica TCS SPE Microsystems Wetzlar GmbH, Wetzlar, Germany) and stacked images were generated in Image J.

### ALP Activity Evaluation

Alkaline phosphatase (ALP) activity was measured spectrophotometrically using a *p*-nitrophenyl phosphate (pNPP) as a substrate for this enzyme (N7653, Sigma Aldrich). ALP activity leads to the conversion of a colorless substrate to the yellow product *p*-nitrophenol. The test was run in triplicates (*n* = 3). 300 μl of ALP substrate was added to each well. Samples were shielded from direct light and incubated for 45 min at room temperature. The entire volume of the solution was then transferred to a new well and 150 μL of 2 M NaOH was added to stop the reaction. Finally, the absorbance of 150 μl of the final solution was measured at 405 nm.

### Immunofluorescence Staining

Scaffolds (*n* = 3) were and washed three times with PBS. Methanol was then added to fix the adhered cells. They were kept for 20 min at room temperature, then stored at −20°C. Scaffolds were washed three times in PBS before staining. After that samples were incubated at room temperature in 0.1% Triton X-100 (T8787, Sigma Aldrich) with 1% BSA (SH30574.02, HY Clone) in PBS. After 30 min was the liquid aspirated and 1% Tween20 (P9416, Sigma Aldrich) in PBS was added. Cells were incubated for 20 min at room temperature. Finally, the samples were washed three times in PBS. Primary antibody rabbit anti-osteocalcin (T4743, Peninsula Laboratories) was diluted in PBS 1:200 and samples were incubated in it overnight at 4°C. In the next step the samples were washed with 0.05% Tween20 (P9416, Sigma Aldrich) in three cycles—after 3, 5 and 10 min of incubation in solution. The samples, after final washing with PBS once a secondary antibody Alexa Fluor 633 (A21070, Life Technologies) in dilution 1:500 in PBS was added, were incubated for 50 min at room temperature in the dark. Samples were then washed three times in PBS. Hoechst 34580 (H21486, Life Technologies) diluted 1:5000 in PBS was added to the samples for 15 min at room temperature. The samples were then washed twice in PBS and scanned under a confocal microscope (Zeiss LSM 880 Airyscan, Germany). It was taken at least four scans from each group. Hoechst 34580 was shown as blue and osteocalcin as red.

### Quantification of Gene Expression Levels

RNA was isolated on day 14 and 28 using a RNeasy Mini kit (Qiagen GmbH, Germany, catalog no. 74106) and following the manufacturer’s protocol. The total amount of nucleic acid was diluted to 366.96 ng/μl for each sample. The reaction was run using triplicates (*n* = 3). The mRNA was subsequently transcribed to cDNA using RevertAid H Minus First Strand cDNA Synthesis Kit (Thermo Fisher Scientific, Waltham, MA, United States, catalog no. K1632), according to the manufacturer’s protocol. Expressions of osteogenic marker genes including runt-related transcription factor 2 (RUNX2) (Hs01047973_m1), collagen type I (COL-I) (Hs00164004_m1), osteocalcin (OC) (Hs01587814_g1), and bone sialoprotein (IBSP) (Hs00913377_m1) (all TaqMan, Thermo Fisher Scientific) were measured. As the 2-ΔCp method was used, the values were normalized with eukaryotic elongation factor-1 (EEF1) (Hs00265885_g1, TaqMan, Thermo Fisher Scientific) as a housekeeping gene. The parameters of qPCR were set as follows: activation—95°C, 10 min; amplification—95°C for 10 s, 60°C for 10 s (50 cycles); termination—40°C for 1 min. The TaqMan Gene Expression Master Mix (Thermo Fisher Scientific, Waltham, MA, United States) and RT-PCR Grade Water (Thermo Fisher Scientific, Waltham, MA, United States) was added to each sample. Samples were stored in a freezer at −80°C between the isolation of RNA, cDNA synthesis, and RT-PCR. To measure the fluorescence intensity Light Cycler 480 (Roche, Basel, Switzerland) was used.

### Quantification of Specific Extracellular Proteins

Medium was collected on day 2, 7, 14, 16, and 21, with four replicates for each group. Medium samples were stored in a freezer at −80°C until final use. Multianalyte profiling of protein levels in the culture medium was performed using the Luminex 200 system (Luminex, Austin, TX, United States), employing xMAP technology where Bone Metabolism Multiplex Assay was used (Human Bone Magnetic Bead Panel, MILLIPLEX, Germany). All the acquired fluorescence data was analyzed by the xPONENT 3.1 software (Luminex, Austin, TX, United States). Selected markers as osteocalcin (OCN), osteopontin (OPN), osteoprotegerin (OPG), dickkopf-related protein 1 (DKK-1), sclerostin (SOST), interleukin-6 (IL-6), and tumor necrosis factor-α (TNF-α) in the culture medium at different time points. All processes were performed according to the manufacturer’s protocols.

### Statistical Analysis

Normality tests were first run on the datasets. Normally distributed results were expressed as means ± standard deviation (SD). One-way ANOVA and Tukey’s tests were used for multiple comparisons among groups while two-way ANOVA and Bonferroni post-tests were applied when different points in time were included. Statistical analysis was run in SPSS12 (IBM SPSS, Armonk, NY, United States). A significant difference was considered where *p* < 0.05, * marking a significant difference versus SBN, # as versus SBP2, and Δ as versus SBP6.

## Results

### Cytotoxicity, Cell Viability/Metabolic Activity, and Cell Proliferation

**FIGURE 2:**
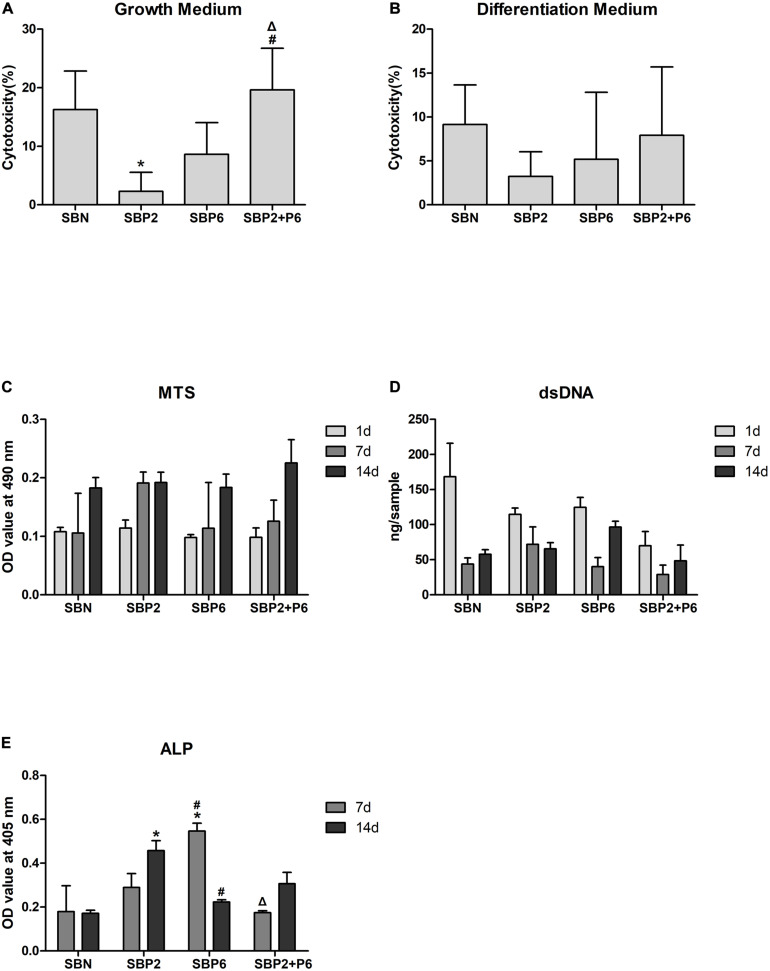
**(A,B)** Cytotoxicity of SBN and SBP analyzed via lactate dehydrogenase (LDH) activity. **(C)** Metabolic activity of hMSCs. **(D)** dsDNA quantification. **(E)** alkaline phosphatase (ALP) activity. *p < 0.05 for when compared to SBN, #*p* < 0.05 for when compared to SBP2, Δ*p* < 0.05 for when compared to SBP6 (*n* = 3).

No cytotoxicity behavior was observed in any of the groups in the growth medium or in the differentiation medium. SBP2 exhibited significantly lower toxicity in the growth environment than the other three groups (Figures 2A,B). The MTS test was used for cell viability evaluation. The cellular metabolism levels of all groups increased during the experiment. No significant difference was detected among groups. A trend of growing metabolic activity could be detected in SBP2+P6, and may portrait a synergistic on cell metabolism (Figure 2C). The amount of dsDNA was also quantified for cell proliferation evaluation. A significant decrease in dsDNA concentration was observed in all groups on day 7. A gradual increase was, however, observed on day 14 for all groups except for the group with SBP2 (Figure 2D). The percentage of stem cells was determined as a number of cells with positive expression of CD105, CD73, CD90 and with negative expression of CD45, CD34, CD14, CD19, HLA-DR. 98.44% of cells in this passage preserved the stemness phenotype (Supplementary Figure A1).

### Cell Expansion

**FIGURE 3:**
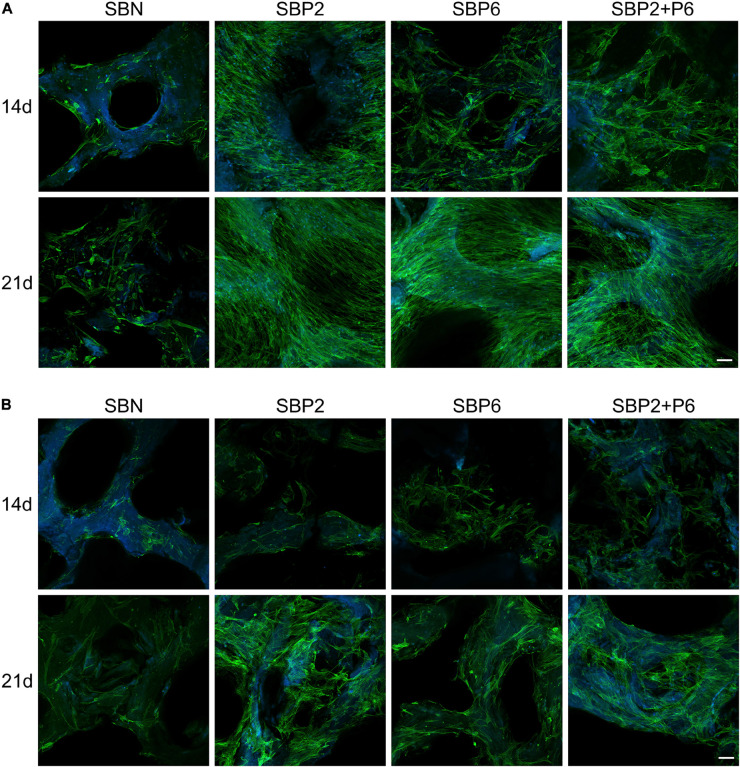
Cell behavior of human mesenchymal stem cells (hMSCs) with growth medium **(A)** and in differentiation medium **(B)** stained with phalloidin (green) and DAPI (blue) observed under laser scanning confocal microscopy. Scale bar: 100 μm (*n* = 3).

Cell behavior on bone grafts was observed under confocal microscopy with phalloidin marking the cytoskeleton and DAPI marking the nucleus. It could be noticed that on day 14, a larger amount of hMSCs was present on SBP than on SBN under the growth environment. Among the SBPs, SBP2 strongly promoted cell proliferation. This was not, however, as strong for SBP6. Multilayers of cells were observed on all SBPs on day 21 (Figure 3A). For cells and bone grafts cultured in differentiation medium, generally cell expansion level was lower than those cultured in growth medium. Nevertheless, SBPs still provided greater cell proliferation effects than SBN (Figure 3B).

### Alkaline Phosphatase Activity

The level of ALP activity in the SBN group was the same on day 7 as it was on day 14. Increased activity was observed in SBP2 and SBP2+P6 in the second week, while contrarily in SBP6 the activity was decreased. The peak was measured on day 7 in SBP6 and significant differences compared to other groups were proved (Figure 2E).

### Immunofluorescence Staining of Osteocalcin

**FIGURE 4:**
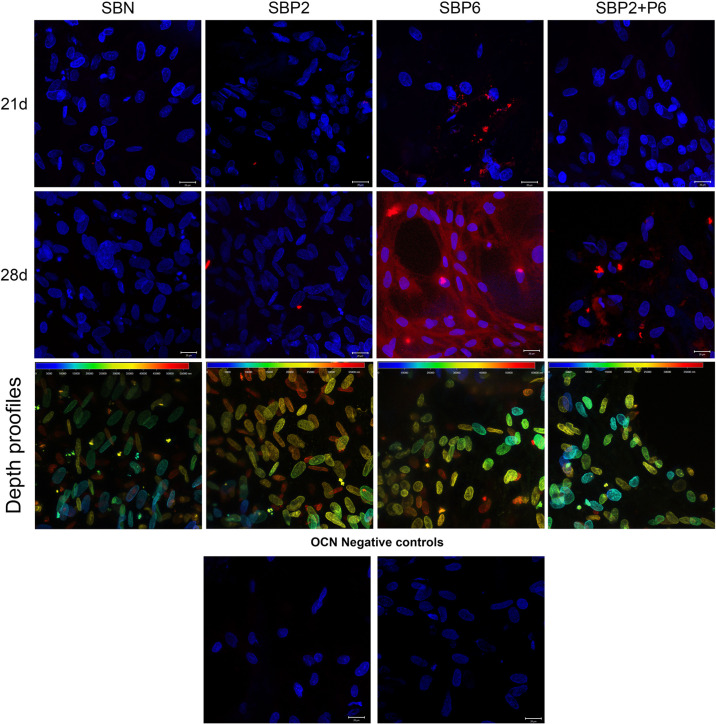
Immunohistochemical staining of OCN (red) and Hoechst 34580 staining of cell nuclei (blue) on day 21 and 28. Cell penetration to the scaffolds are presented as depth profiles at day 28 in third row and OCN negative controls at the bottom. Scale bar: 20 μm (*n* = 4).

The expression level of osteocalcin (OCN) was detected via the immunofluorescence staining method. A high level of OCN was detected in SBP6 on day 28. The expression of OCN could also be noted in P6 on day 21 and in SBP2+P6 on day 28 (Figure 4). The dept profiles shows that the cells penetrated deep into the porous materials (Figure 4). Due to the negative controls, we can exclude possible non-specific interaction of secondary antibody with the biomaterial or biomaterial autofluorescence.

### Gene Expression

**FIGURE 5:**
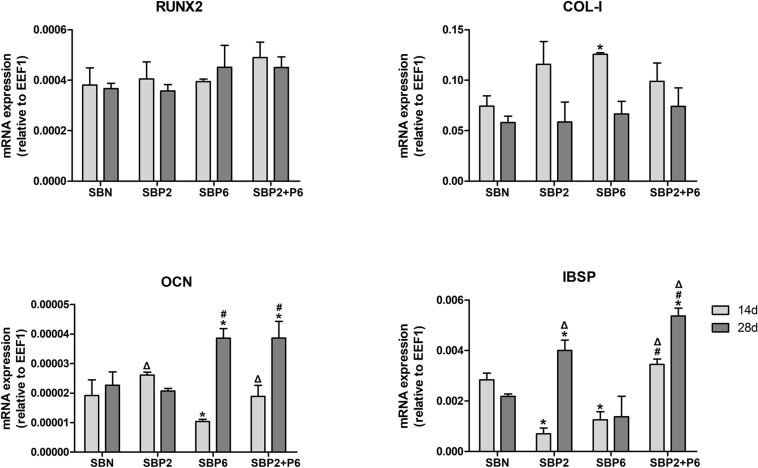
Osteogenic related gene expression analyzed via qPCR. *p < 0.05 for when compared to SBN, #*p* < 0.05 for when compared to SBP2, Δ*p* < 0.05 for when compared to SBP6 (*n* = 4).

Expressions of osteogenic marker genes including runt-related transcription factor 2 (RUNX2), collagen type I (COL-I), osteocalcin (OCN), and bone sialoprotein (IBSP) were measured on day 14 and 28. No difference was observed for RUNX2 among all groups. The COL-I expression in SBP6 was significantly higher than in SBN on day 14. SBP6 exhibited the lowest OCN expression on day 14, while SBP6 and SBP2+P6 were significantly higher than other groups on day 28. As to IBSP, SBN and SBP2+P6 displayed higher expressions than SBP2 and SBP6 on day 14, whereas SBP2 and SBP2+P6 were higher than others on the day 28, with SBP2+P6 as the highest (Figure 5).

### Quantification of Specific Extracellular Proteins

On day 2, 7, 14, 16 and 21, medium was collected and multianalyte profiling of protein levels in the culture medium was performed including osteocalcin (OCN), osteopontin (OPN), osteoprotegerin (OPG), dickkopf-related protein 1 (DKK-1), sclerostin (SOST), IL-6, and TNF-α. Significant differences in OCN secretion could be observed in both the growth and osteogenic environment. In the growth environment, SBPs all exhibited higher levels of OCN compared to SBN on the 7th day, and this remained high on the 21st day in SBP6 and SBP2+P6. With stimulation of osteogenic medium, secretion of OCN in SBPs was kept high during all the experiment. Significant differences could be detected in all SBPs on day 7 and 14, and in SBP6 and SBP2+P6 on day 21 compared to SBN.

**FIGURE 6:**
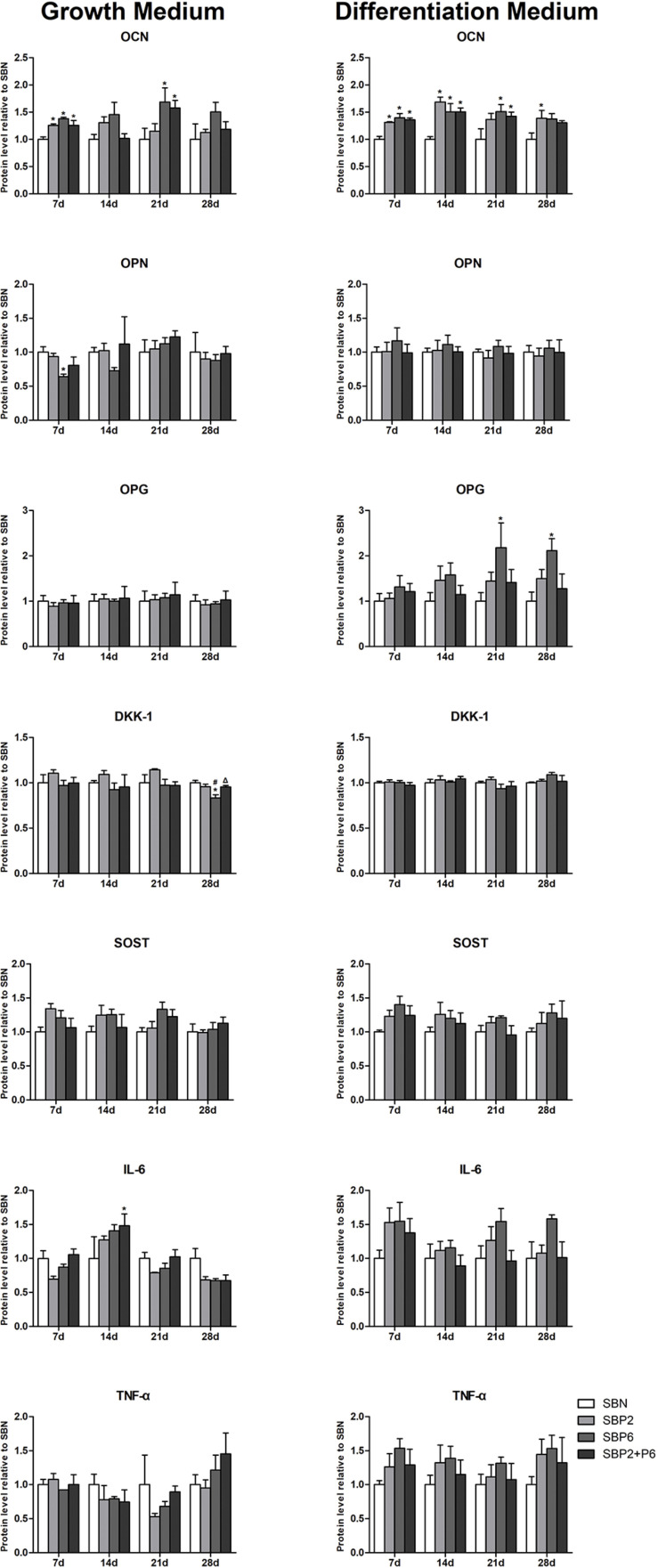
The quantification results of specific extracellular protein (OPN, OPG, DKK-1, SOST, IL-6, and TNF-α). Results were shown normalized to SBN at different time points. *p < 0.05 for when compared to SBN, #*p* < 0.05 for when compared to SBP2, Δ*p* < 0.05 for when compared to SBP6 (*n* = 4).

For OPN, significant difference was only noted on day 7 through comparing SBP6 with SBN in the growth environment. No other difference was detected. For OPG, SBP6 exhibited higher secretion on day 21 and 28 than SBN in the osteogenic environment. For DKK-1, secretion level was at the lowest in SBP6 compared to all other groups on the 21st day in growth environment. For SOST, not significant difference was found among all groups in the two different environments. As to the inflammatory markers, significant difference could only be detected on day 14 in IL-6 when comparing SBP2+P6 and SBN (Figure 6).

## Discussion

In this study, we utilized hMSCs to study the biocompatibility and biological functions of xeno-hybrid bone graft with entrapped biomimetic peptides. The cell behavior was investigated in both growth and osteogenic microenvironments. The biomimetic synthetic peptides (P2 and P6) belongs to a group named IDPs which has no definite three-dimensional structure under natural conditions (Kalmar et al., 2012; Wright and Dyson, 2015). These biomimetic IDPs enjoys unique benefits in combination, spatial transformation, and coordination. IDPs are involved in plenteously of biological activities and are regarded as “one too many signaling” protein. Hydroxyapatite (HAp) crystal formation, growth control, and its orientation is controlled partly of fully disordered IDPs (Wald et al., 2017). P2 and P6 has common proline-rich regions to such biomineralization controlling (amelogenin and ameloblastin) (Ramis et al., 2012). The idea behind the studied bone graft substitute was to pool a commercially bone graft (SmartBone^®^) with a biomimetic peptide with IDP functions. The request for a more sophisticated bone graft with advanced bioactivity and biomimetic properties is growing as orthopedic surgeons are using BGs in more challenging clinical cases than earlier (Haugen et al., 2019; Sanz et al., 2019). Although variances of biomolecule stimuli, such as recombinant growth factors have been successfully embedded in other bone graft materials for promoting osteogenic differentiation or enhancing osseointegration, these biomolecules are associated with both high costs and lack of sufficient evidences of safety and efficacy (Fu et al., 2013; James et al., 2016), which impedes long-term clinical applications. Carragee et al. found that the risk of adverse dealings associated with rhBMP-2 was 10–50 times greater than what was originally reported (Carragee et al., 2011). However, synthetic biomimetic peptides are both safer and more cost-effective (Delawi et al., 2016), and in addition, they can withstand exposure to strong organic solvents and ethylene oxide sterilization (Zhu et al., 2020). When using growth factor such as BMPs ones need to be precaution with exposure to organic solvents which limits its use for in new biomaterials.

The addition of the biomimetic P2 and P6 improved the biological characters of SBN (Roato et al., 2018). As shown in confocal images for growth medium, SBPs had a strong effect on promoting the proliferation of attached stem cells when compared with SBN, particularly SBP2, where multilayers of cells were already formed at the early stage (day 14), which was also in correspondence with the result of LDH activity, indicating that SBPs could provide an ideal microenvironment for hMSCs colonization. Mesenchymal stem cells are multipotent, active as forerunners to a several cell types such as adipocytes, osteoblasts, and chondrocytes. When the hMSCs were exposed to the osteogenic differentiation medium, the proliferation would be partly suppressed and hMSCs would differentiate into osteoblasts phenotype. However, even suppressed, large numbers of cells could still be observed on the surface of SBPs. The proliferation progress was not totally inhibited in the differentiation environment under the stimulation of IDPs.

A series of experiments was performed to study how the IDPs affected the differentiation of hMSCs on the bone grafts. ALP activity is an early marker of osteoblast differentiation. SBP6 exhibited a strong effect on ALP activity enhancement at an early point in time (day 7), suggesting that P6 might potentially be involved in this progress of the osteogenesis effect. An interesting phenomenon occurred for ALP activity where we saw a higher expression for SBP2 than the combination of both peptides (SBP2+P6). It seemed that SBP6 had higher stimulation on ALP activity on day 7, while SBP2 had higher stimulation on ALP activity on day 14. When P2 and P6 release together into the cell media, we observed a halved effects on each time points, leading to a lower ALP activity instead of synergistic effect. We therefore postulate that the P2 and P6 might have competitive effect on their receptors. Further analysis of the mRNA expression revealed that SBP6 could affect the expression of COL-I on the 14th day, and also demonstrated that OCN expression was strongly promoted on the 28th day in SBP6 and SBP2+P6, while SBP2 could also stimulate OCN expression on the earlier time point (day 14). In addition, P2 could promote IBSP expression, as proved by the results of SBP2 and SBP2+P6 on day 28. The expression of RUNX2 mRNA, which is a transcription factor at the beginning of the osteogenic signaling cascade, showed no significant differences. We assumed that OCN was the key point of the phenomenon. OCN, a bone γ-carboxyglutamic acid protein, is a secreted factor influencing matrix mineralization and global metabolism. It is a small protein that composes approximately 10% of non-collagenous protein part in bone (Gallop et al., 1980). OCN is associated with the mineralized matrix of bone and has the ability to bind calcium, showing an adsorption affinity for hydroxyapatite (McKee and Cole, 2012). Therefore, the addition of IDPs in this xeno-hybrid bone graft could facilitate extracellular matrix mineralization potentially via boosting OCN synthesis. Similarly, osteogenic peptides derived from collagen type I, BMP-7 and mainly BMP-2 enhanced OCN expression and synthesis in a dose-dependent manner (Lukasova et al., 2017). Since OCN is synthesized almost exclusively by highly differentiated osteoblasts (Price et al., 1976; Eastell and Hannon, 2007)[Bibr B10][Bibr B36], these results indicated that the hMSCs on the SBPs were in the process of differentiation to osteoblasts phenotype. The effect on OCN was also seen in a previous study with these materials, when human osteoblasts from a variety of donors were seeded onto SmartBonePep^®^ (Zhu et al., 2020).

Luminex methods allowed for further analyze the osteogenesis markers on the protein level. DKK-1 and SOST are involved in the down regulating of osteogenesis. No difference was found among groups in two different culturing environments for these two markers, demonstrating that there was no suppressing effect for SBN nor SBPs. For the inflammatory markers, only on the 14th day we could notice that SBP2+P6 secreted a higher amount of IL-6 than SBN. This indicated that the combination of these two peptides might have slightly up-regulated inflammation effects. OPN and OPG are important regulatory factors for osteogenesis. However, few differences were noticed in this study. Nevertheless, hMSCs on SBP6 still secreted a higher amount of OPG when exposed to the differentiation environment. What was exciting was the results of OCN secretion quantification: SBPs intensively enhanced the production of OCN at each point in time compared with SBN when hMSCs were cultured in differentiation medium. This effect could still, although reduced, be detected when the differentiation environment was switched to the growth environment. These results demonstrated that SBPs, especially with P6 entrapped, could promote OCN secretion in hMSCs even without the stimulation from the osteogenic medium. SBP6 therefore has an osteogenic effect on hMSCs mainly by facilitating the OCN expression. Finally, the immunofluorescence results again provided confirmation of this effect, a high amount of OCN being noticed on the cells attached to SBP6. A limitation of our study was the bone graft was only exposed to *in vitro* conditions. In a clinical setting, the cell attached to the bone grafts can act slightly different due to various stimuli including blood and lymph flow which we are not able to mimic in static cultivation. However, *in vitro* experiments are crucial first step in any new material development.

Above all, the entrapped biomimetic peptide modified the biological functions of SmartBone^®^, promoting the proliferation and osteogenesis process of hMSCs. Specifically, SBP6 promoted osteogenesis partially via upregulating OCN, and SBP2 tended to facilitate cell proliferation.

## Conclusion

Two biomimetic peptides, P2 and P6, when in corporate into a degradable bone graft, demonstrated to possess multi-modal biological regulations on hMSCs. The biomimetic peptides improved the bone formation capacities bioactive of mesenchymal stem cells and these peptide withstood the bone graft manufacturing processes including exposure to strong organic solvents and ethylene oxide sterilization. P6 increased stem cell attachment and proliferation when compared to P2 and controls (bone graft without biomimetic peptides). The studied biomimetic peptides may be a future candidate for enhancing the clinical performance of bone graft.

## Data Availability Statement

The raw data supporting the conclusions of this article will be made available by the authors, without undue reservation.

## Author Contributions

HZ and VB contributed equally to this work. VB, GP, FB, SL, and HH performed conceptualization and methodology. FB performed sample preparation. HZ and VB performed data curation and experimental work. HZ, VB, and HH performed writing and original draft preparation. JX, GP, EF, SL, and HH performed supervision, reviewing, and editing. All authors contributed to the article and approved the submitted version.

## Conflict of Interest

The rights for SmartBone^®^ and SmartBonePep^®^ patents and trademarks belong to Industrie Biomediche Insubri S.A. (Switzerland). GP is a founding shareholder and the executive vice president of Industrie Biomediche Insubri S.A. (Switzerland), the company that fully owns all IPRs (US Patent US8367602B2, PCT/IB2007/004068, EP Patent EP2358407B1, and related bundles) on SmartBone^®^ and SmartBonePep^®^ and their trademarks. FB works as employee for the same company. FB was employed by the company Helmholtz Zentrum München Deutsches Forschungszentrum für Gesundheit und Umwelt (GmbH). HH and SL was part time employed by the company Corticalis AS. The remaining authors declare that the research was conducted in the absence of any commercial or financial relationships that could be construed as a potential conflict of interest.
