# Supplementary material for: Xeno-Hybrid Bone Graft Releasing Biomimetic Proteins Promotes Osteogenic Differentiation of hMSCs
Source: Front Cell Dev Biol. 2020 Dec 22;8:619111. doi: 10.3389/fcell.2020.619111 (PMC7784409; doi:10.3389/fcell.2020.619111)
Supplement: Supplementary file 1 [file Table_1.DOCX]

Supplementary Material


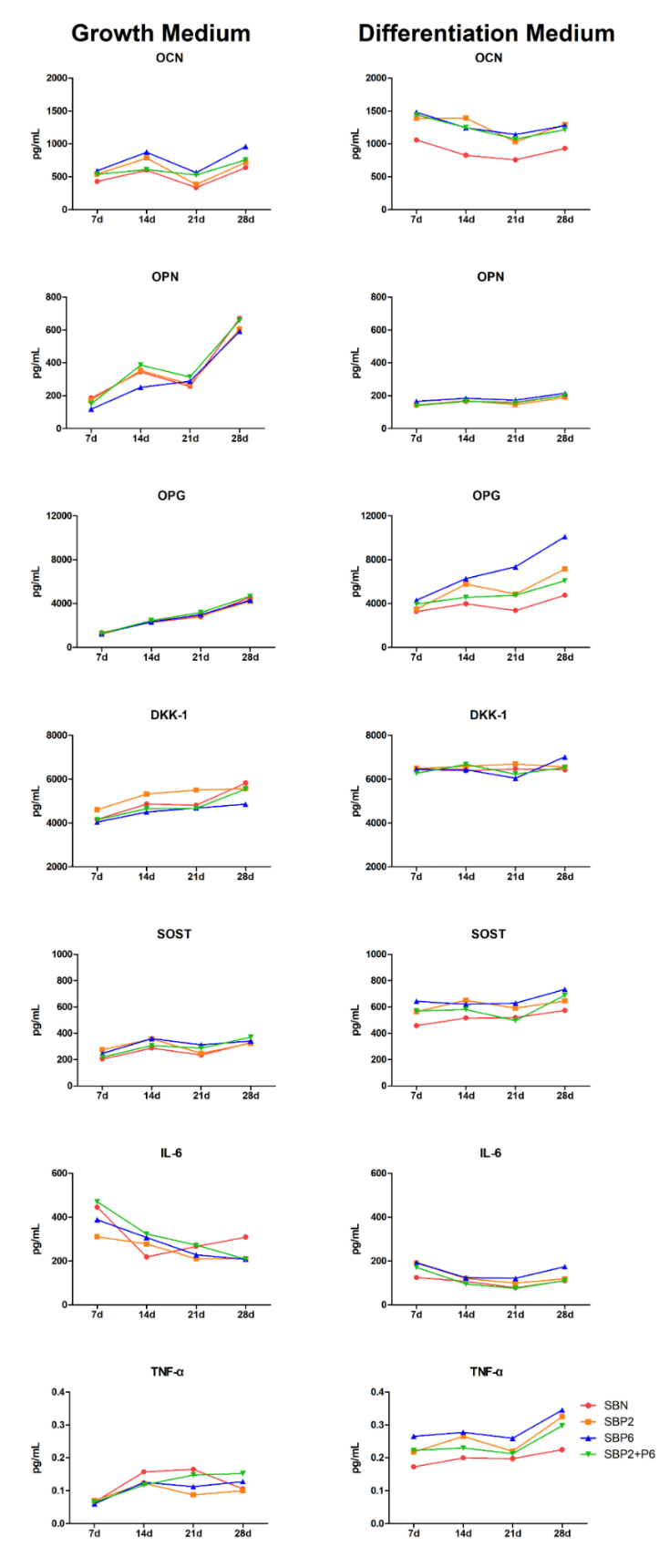


**Figure A1.** Quantification results of specific extracellular proteins (OCN, OPN, OPG, DKK-1, SOST, IL-6 and TNF-α). Results were shown as lines and scatters.


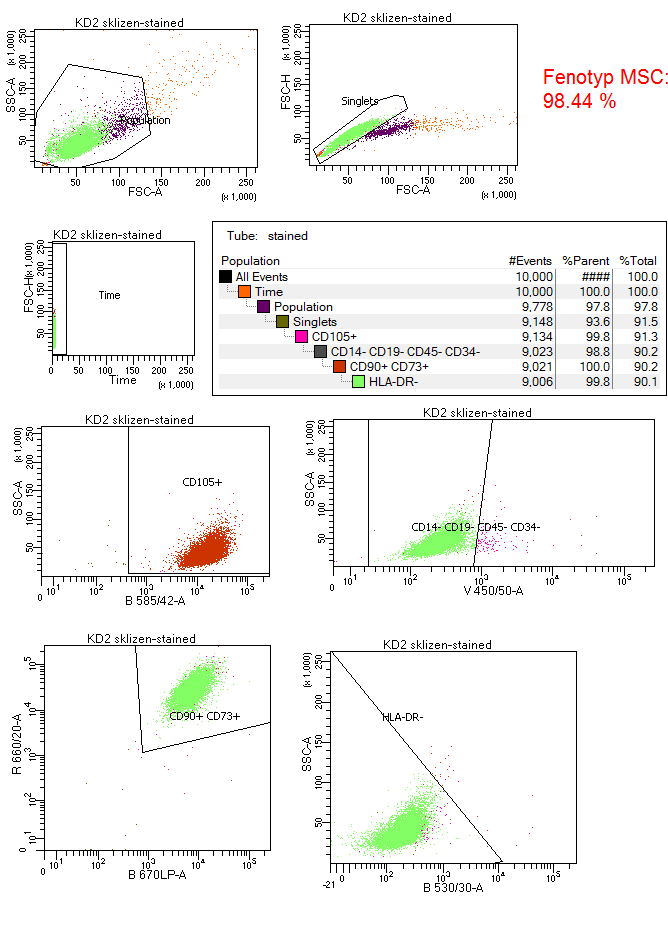


**Figure A2.** The percentage of stem cells is determined as a number of cells with positive expression of CD105, CD73, CD90 and with negative expression of CD45, CD34, CD14, CD19, HLA-DR. 98.44 % of cells in this passage preserved the stemness phenotype.
